# Supplementary material for: Scaling models of visual working memory to natural images
Source: Commun Psychol. 2024 Jan 3;2:3. doi: 10.1038/s44271-023-00048-3 (PMC11332237; doi:10.1038/s44271-023-00048-3)
Supplement: Supplementary file 1 — Supplementary Information [file 44271_2023_48_MOESM1_ESM.pdf]

Supplementary Information

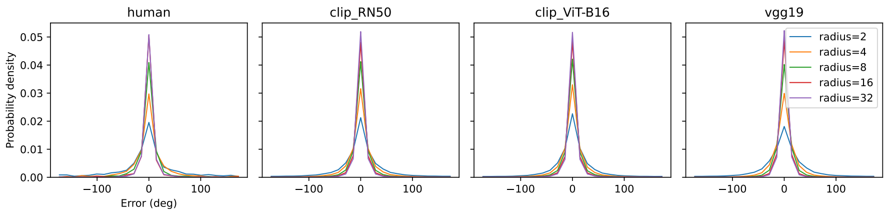

Fig. S1 Histogram estimates of error distributions for models and humans in Scene Wheels dataset. Each wheel radius is plotted as a separate line.

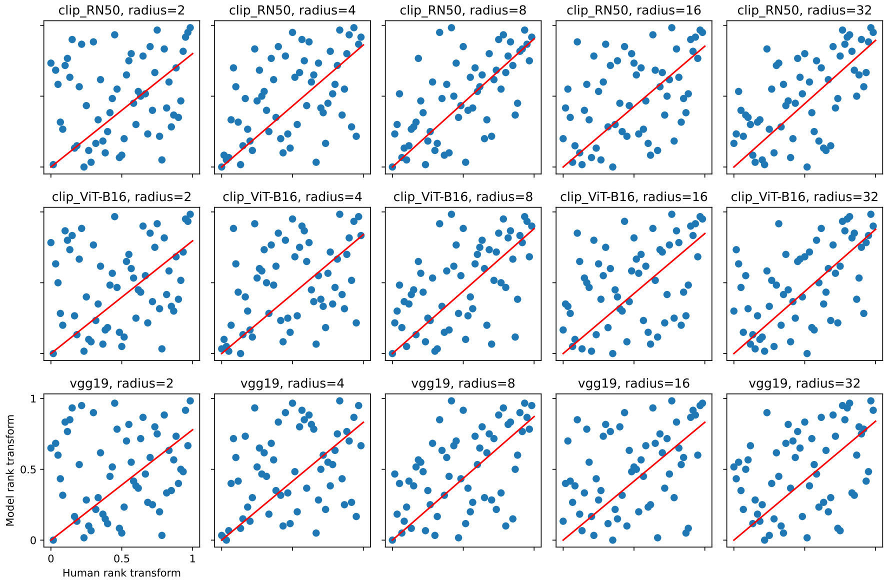

Fig. S2 Scatter plot of the (normalized) rank-transformed data in Scene Wheels experiment. Each data point was transformed from an error magnitude to its rank relative to others. Each point corresponds to a 30 degree bin of the response wheel, within a particular scene wheel and a particular radius. As in all other analyses, human and model errors were first averaged within these bins. Red lines are least-squares fit with fixed zero intercept.

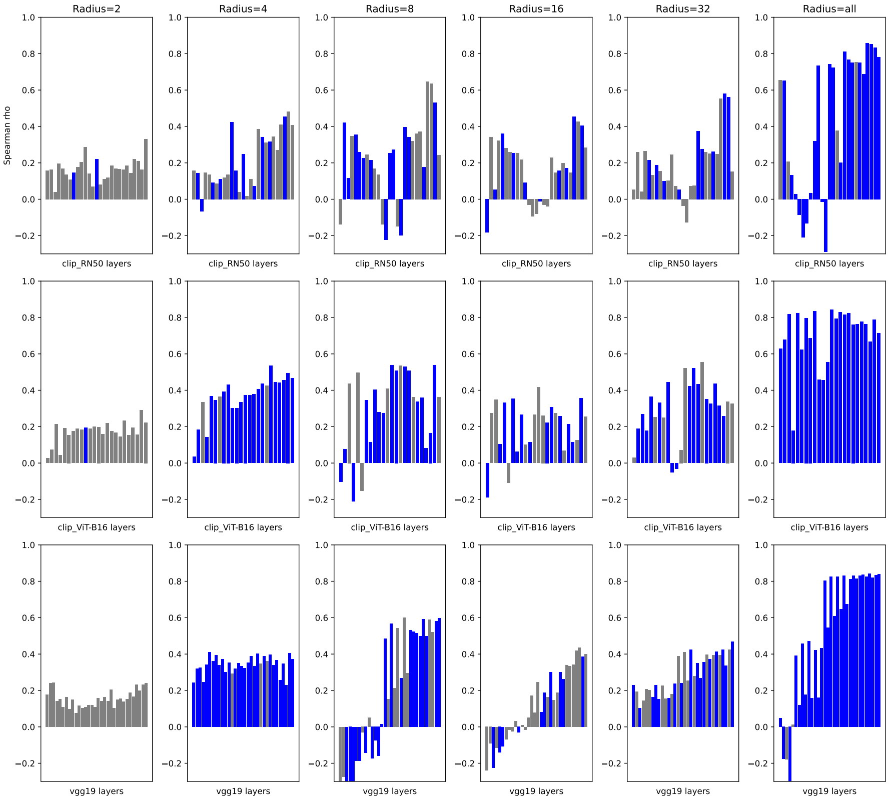

Fig. S3 Spearman rank correlations for trial difficulty between each layer in selected DNN architectures and humans on Scene Wheels dataset. Blue bars indicate p-values less than 0.05.

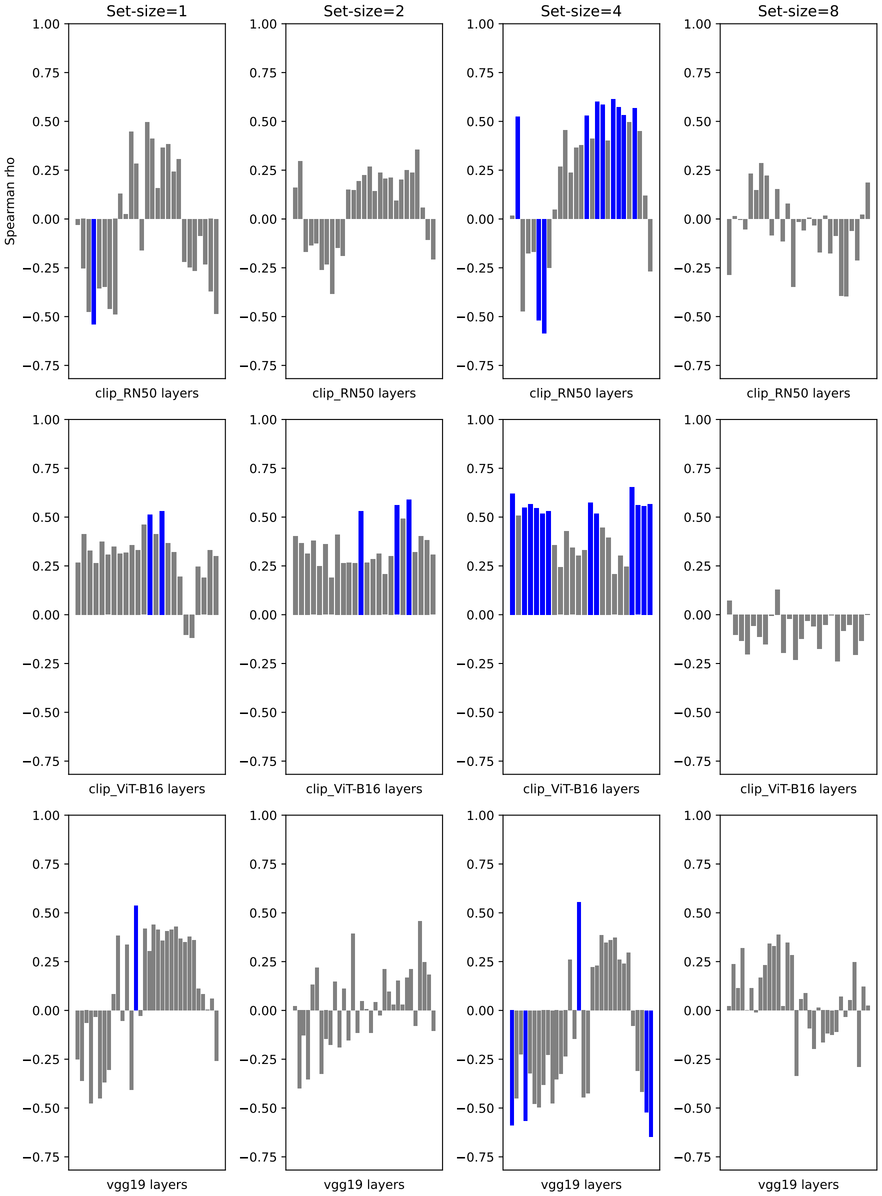

Fig. S4 Spearman rank correlations for trial difficulty between each layer in selected DNN architectures and humans on orientation memory dataset. Blue bars indicate p-values less than 0.05.

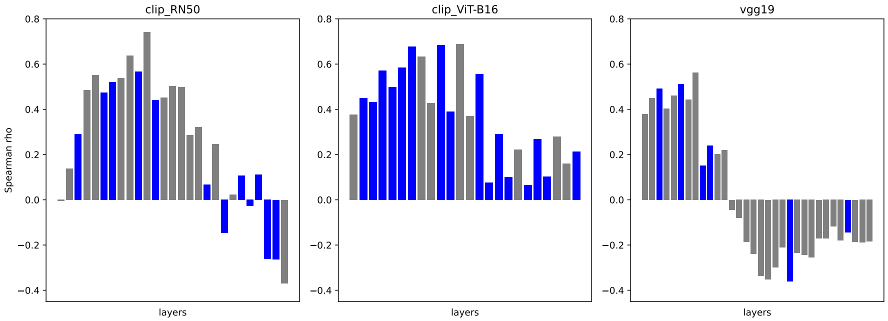

Fig. S5 Spearman rank correlations for trial difficulty between each layer in selected DNN architectures and humans on color memory dataset. Blue bars indicate p-values less than 0.05.

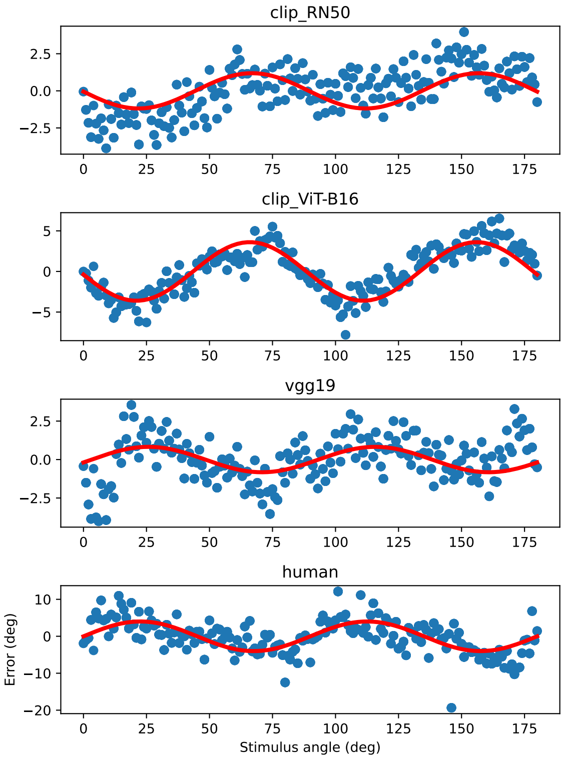

Fig. S6 Result of repeating analysis in Fig. 6 fitting to all set-sizes rather than just set-size 1. Note that the CLIP ResNet-50 panel is the only one that differs from its counterpart in the main text.
